# Inhibition of DNA–Topoisomerase I by Acylated Triterpene Saponins from *Pittosporum angustifolium* Lodd.

**DOI:** 10.1007/s13659-016-0087-5

**Published:** 2016-01-23

**Authors:** Christian Bäcker, Malgorzata N. Drwal, Robert Preissner, Ulrike Lindequist

**Affiliations:** Department of Pharmaceutical Biology, Institute of Pharmacy, Ernst Moritz Arndt University Greifswald, Friedrich-Ludwig-Jahn-Straße 17, 17489 Greifswald, Germany; Structural Bioinformatics Group, Institute for Physiology, Charité – University Medicine Berlin, Lindenberger Weg 80, 13125 Berlin, Germany

**Keywords:** *Pittosporum angustifolium*, Acylated triterpene saponins, Cytotoxicity, Topoisomerase I, Docking

## Abstract

**Abstract:**

Previous phytochemical investigation of the leaves and seeds of *Pittosporum angustifolium* Lodd. led to the isolation and structural elucidation of polyphenols and triterpene saponins. Evaluation for cytotoxicity of isolated saponins revealed that the predominant structural feature for a cytotoxic activity are acyl substituents at the oleanane aglycon backbone. The present work reports the results of a screening of 10 selected acylated saponins for their potential to inhibit the human DNA-topoisomerase I, giving rise to IC_50_ values in a range of 2.8–46.5 µM. To clarify the mode of observed cytotoxic action and, moreover, to distinguish from a pure surfactant effect which is commonly accompanied with saponins, these results indicate an involvement of the topoisomerase I and its role as a possible target structure for a cytotoxic activity. In addition, computational predictions of the fitting of saponins to the topoisomerase I–DNA complex, indicate a similar binding mode to that of clinically used topoisomerase I inhibitors.

**Graphical Abstract:**

Ten acylated triterpene saponins from *Pittosporum angustifolium* were investigated for their potential to inhibit the human DNA-topoisomerase I and computational predictions of the fitting of saponins to the topoisomerase I–DNA complex were carried out.
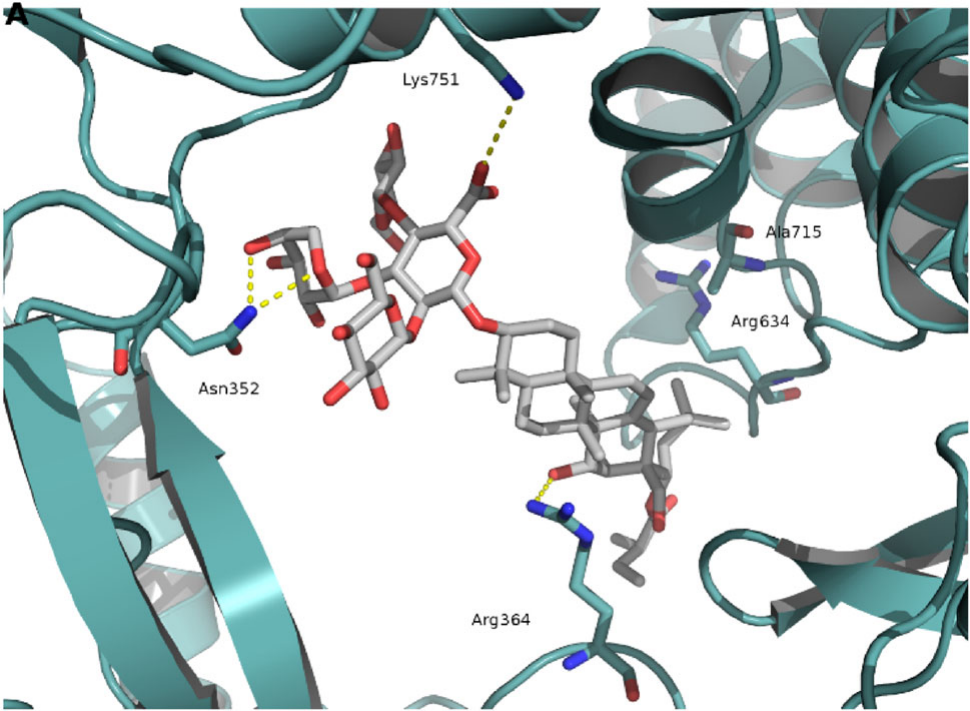

## Introduction

*Pittosporum angustifolium* Lodd. (Pittosporaceae) is a small tree distributed in almost all inland areas of Australia. Different remedies prepared from aerial parts of the plant are used in the field of traditional Aboriginal medicine e.g. for the treatment of skin diseases and cough and, furthermore, as a supportive agent for complementary therapy strategies [1, 2]. Recently, we have extensively investigated the phytochemistry of the seeds and leafs of *P. angustifolium*, resulting in the isolation and structural elucidation of five known polyphenols (quercetin glycosides and dicaffeoylquinic acids) and 33 mono- and bisdesmosidic triterpene saponins, of which 29 were reported for the first time, possessing oleanane, rare 17,22-seco-oleanolic acid and taraxastane aglycones [3–7]. Since the biological activities of triterpene saponins are described as manifold as their number of possible structures [8], often a generalized cytotoxic activity but also detailed modes of action, as well, are published [9]. In recent studies we have screened isolated compounds of *P. angustifolium* for their cytotoxic potential against three tumorigenic cell lines (MCF7—human breast cancer, 5637—human urinary bladder carcinoma, LN18—human glioblastoma) and one non-tumorigenic cell line (HaCaT—human keratinocyte) [3, 5, 7, 10]. We found, that monodesmosidic saponins of the oleanane-type, featuring acyl-substituents like acetyl (Ac), 2-acetoxy-2-methylbutyroyl (AcOMebu), angeloyl (Ang) or 2-methylbutyroyl (Mebu) groups at C-21/C-22 of the aglycone backbone showed cytotoxic activity up to the low µM-range [3, 5, 10]. Similar results concerning those structural elements of investigated saponins combined with a linked cytotoxicity have also been described in the past [11–15]. To understand the possible mechanisms of cytotoxic and antiproliferative effects, a literature search for structural analogues has drawn our attention to the topoisomerase enzymes. For example, naturally occurring topoisomerase I and/or II inhibitors were found among the structural classes of alkaloids, flavonoids, naphthoquinones, di- and triperpenes [16–19], while literature data for triterpene saponins is barely represented [15, 20]. Wang et al. [15] discovered, that saponins, exhibiting high structural similarity to the here investigated compounds, had no influence on topoisomerase II. Instead, an inhibition of topoisomerase I has been observed, while the aglycones of active glycosides were without discernible impact. Encouraged by these outcomes, herewith we report the results of an investigation of 10 acylated triterpene saponins (Table 1) isolated from *P. angustifolium* (**1**-**10**) [3, 5, 6] and partially from other *Pittosporum* species before (**4**, **8**, **9**) [21, 22] for their potential to inhibit the human topoisomerase I via gel based relaxation assay. We further present computational predictions of the binding mode of the active compounds within the topoisomerase I–DNA complex.

**Table 1:** Cytotoxicity and inhibition of human topoisomerase I by acylated triterpene saponins from *Pittosporum angustifolium* 
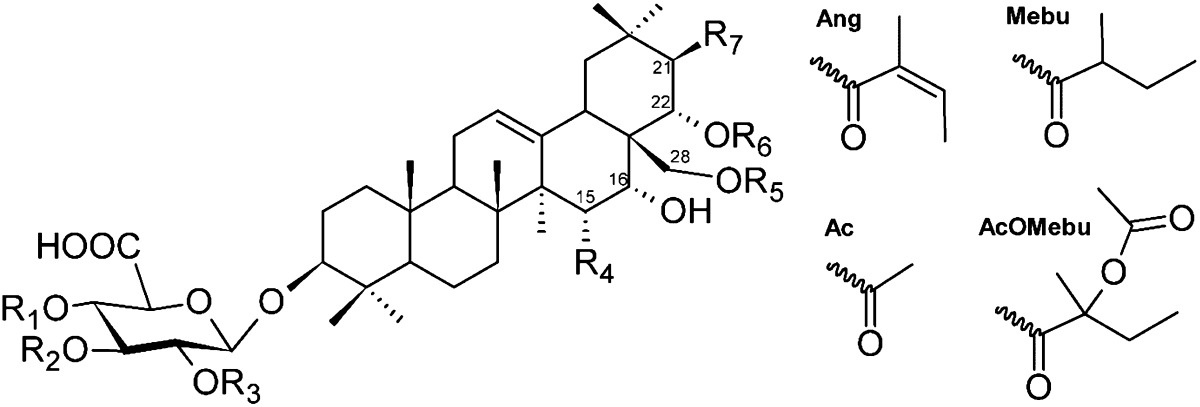

No./name	R_1_	R_2_	R_3_	R_4_	R_5_	R_6_	R_7_	IC_50_ [µM] Topo I^a^	IC_50_ [µM] Cytotoxicity^b^			
									5637	MCF-7	LN-18	HaCaT
**1**/pittangretoside A^b^	Ara (f)	Ara (p)	Glc	OH	H	Ang	H	7.5 ± 0.6	4.1 ± 1.0	25.7 ± 1.9	7.6 ± 1.7	15.2 ± 0.9
**2**/pittangretoside D^b^	H	H	[Rha-(1 → 2)]-Gal	OH	H	Ang	H	4.8 ± 2.4	17.9 ± 1.0	36.6 ± 5.7	35.7 ± 5.2	>118^c^
**3**/pittangretoside F^b^	Ara (f)	Ara (p)	Glc	OH	Mebu	H	H	16.8 ± 0.4	> 106^c^	n.t.	n.t.	n.t.
**4**/IIIA_3_ [21]	Ara (f)	Ara (p)	Glc	OH	H	Ac	OAng	8.6 ± 0.1	7,6 ± 1.6	19.8 ± 2.2	13.4 ± 0.8	15.9 ± 1.3
**5**/pittangretoside N^b^	Ara (f)	Ara (p)	Glc	OH	H	Ang	OAc	6.5 ± 0.6	13,1 ± 1.0	28.7 ± 3.6	10.4 ± 2.7	34.1 ± 2.8
**6**/pittangretoside O^b^	Ara (f)	Ara (p)	Glc	OH	H	AcOMebu	OAc	7.5 ± 1.6	9,0 ± 1.4	22.9 ± 1.8	4.4 ± 0.7	19.2 ± 2.1
**7**/pittangretoside P^b^	Ara (f)	Ara (p)	Glc	OH	H	Ac	OMebu	4.2 ± 0.2	5,6 ± 1.2	28.7 ± 3.2	7.0 ± 0.9	11.2 ± 0.5
**8** [22]	Ara (f)	Ara (p)	Glc	OH	H	Mebu	H	2.8 ± 1.1	n.t.	n.t.	3.9 ± 0.1	n.t.
**9**/IIIC_4_ [21]	Ara (f)	Ara (p)	Glc	OH	H	Ang	OAng	46.5 ± 11.5	2,2 ± 0.2	8.9 ± 0.8	4.6 ± 0.5	2.2 ± 0.5
**10**/pittangretoside T^b^	Ara (f)	Ara (p)	Gal	H	H	Ang	OAng	5.0 ± 3.3	n.t.	n.t.	n.t.	n.t.
Camptothecin (positive control)								7.4 ± 1.5	n.t.	n.t.	n.t.	n.t.

*Ara* arabinose,* f/p* furanose/pyranose,* Glc* glucose,* Gal* galactose,* Rha* rhamnose,* Ang* angeloyl,* Mebu* 2-methylbutyroyl,* Ac* acetyl,* AcOMebu* 2-acetoxy-2-methylbutyroyl;* n.t.* not tested

^a^ ±SD, n = 2

^b^ ±SD according to [3, 5, 10], 5637: human urinary bladder carcinoma cells, MCF-7: human breast cancer cells, LN-18: human glioblastoma cells, HaCat: human keratinocyte cells

^c^ no cytotoxic activity up to 125 µg/ml

## Results and Discussion

**Fig. 1:**
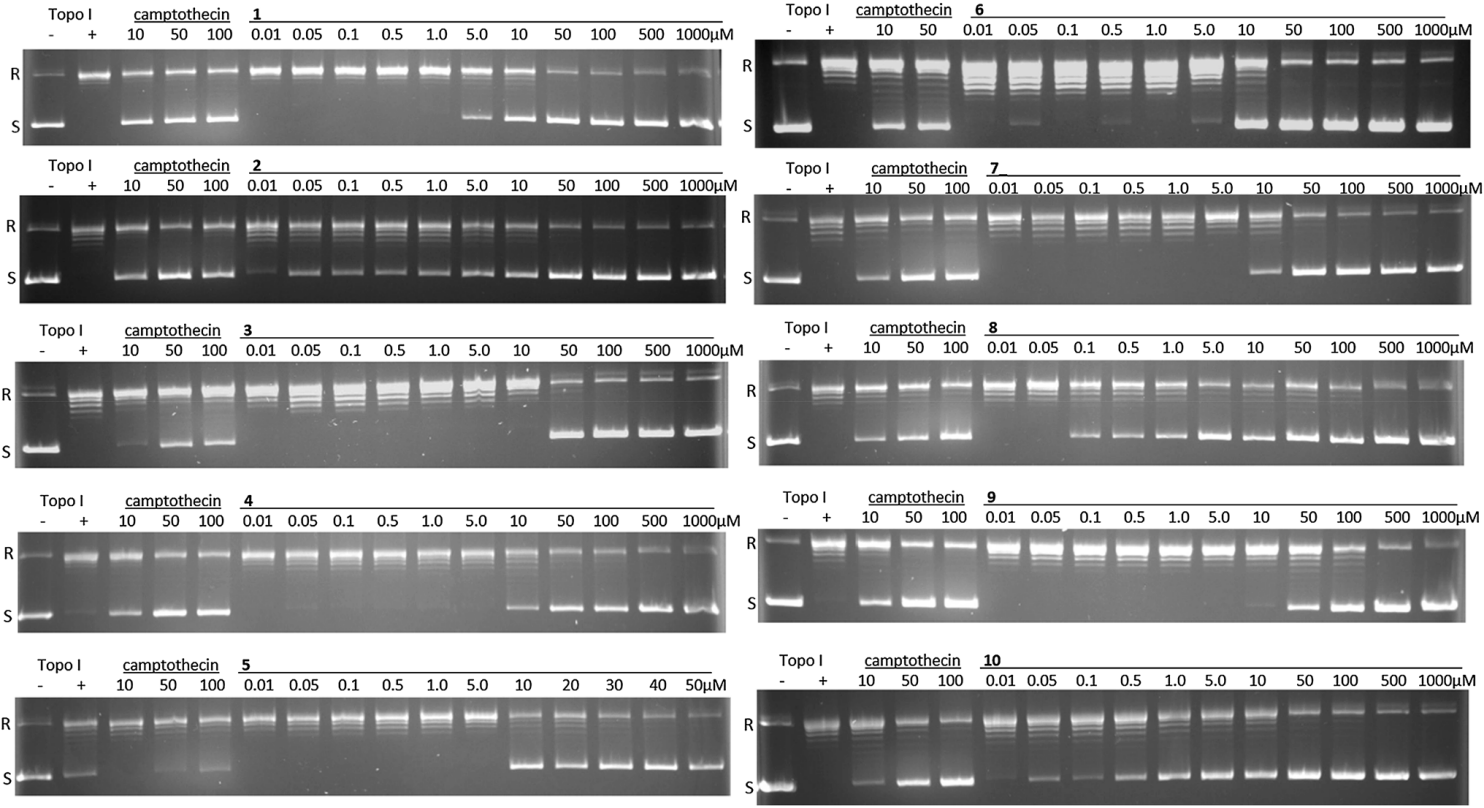
Activity of acylated triterpene saponins **1**-**10** on topoisomerase I mediated relaxation (R) of supercoiled pBR322 DNA (S) in a dose dependent manner. Gels were stained with ethidium bromide. Camptothecin was used as positive control. Controls topo I (−) included supercoiled DNA without enzyme, topo I (+) contained supercoiled DNA in the presence of enzyme. For each substance (**1**-**10**), one of the two independent experiments is shown

For all triterpene saponins from *Pittosporum angustifolium* (**1**-**10**), acylated at C-21/C-22 (**1**, **2**, **4**-**10**) or rather at C-28 (**3**), the inhibition of human topoisomerase I was determined in a DNA relaxation assay. IC_50_ values in a range of 2.8–46.5 µM were generated (Table 1; Fig. 1). Most compounds (**1**, **2**, **4-8** and **10**) showed activities comparable to or higher (2.8–8.6 µM) than that of the positive control camptothecin (7.4 µM), while saponins **3** and **9** were slightly less active with IC_50_s of 16.8 and 46.5 µM, respectively. Also the only tested non-cytotoxic structure **3** [10], whose 2-methylbutyroyl acyl residue is attached to C-28, caused an inhibition of topoisomerase I which was relatively weaker, whereas **9**, the one possessing high cytotoxicity for all investigated cell lines [5, 10] showed the weakest activity with 46.5 µM. Interestingly, compound **10**, possessing no hydroxyl group at C-15 and a galactose moiety instead a glucose unit as in **9**, showed a nearly tenfold stronger inhibition (5.0 µM) than compound **9**. The most potent inhibitor with an IC_50_ of 2.8 µM turned out to be compound **8**, carrying a 2-methylbutyroyl acyl residue at C-22 and found out to be the most cytotoxic compound tested on LN18 cells [5, 10]. Moreover, compound **8** exhibited a more potent inhibition than structure **1**, possessing an angeloyl moiety (**1**) instead a 2-methylbutyroyl residue (**8**). A finer distinction between the different patterns of acylation at C-22/C-21 as seen for the cytotoxicity screening [5, 10], from which more obvious tendencies or conclusions could be summarized, have not been clearly observed in the results of the topoisomerase I assay. Nevertheless, the present data indicate that certain compounds possess pronounced cytotoxic effects and a serious inhibition of topoisomerase I as well (compounds **1**, **2**, **4-8**), but also contradictory relationships (compounds **3**, **9**) were observed. As a common feature of investigated structures **1**-**10**, all of them possess at least one 5-carbon acyl substituent with a functional element of a keto group, in detail an angeloyl, 2-methylbutyroyl or a partially modified derivative like a 2-acetoxy-2-methylbutyroyl residue, either at C-28 (**3**), C-22 (**1**, **2, 5, 6, 8**), C-21 (**4**, **7**) or at both, C-21 and C-22 (**9**, **10**). Furthermore, acetyl groups can additionally be attached at C-22 (**4**, **7**) or at C-21 (**5**, **6**). As already mentioned, those acyl residues seem to play an essential key role for cytotoxicity and, beyond, we could substantiate their importance for an inhibition of the topoisomerase I as recently reported [15]. On top of that, sugar substitution patterns as well as the physio-chemical environment in close proximity to the acyl residues (hydroxy group at C-15 vs. no substitution as seen for **10** and **9**) seem to trigger a de- or increase of an inhibitory activity.

**Fig. 2:**
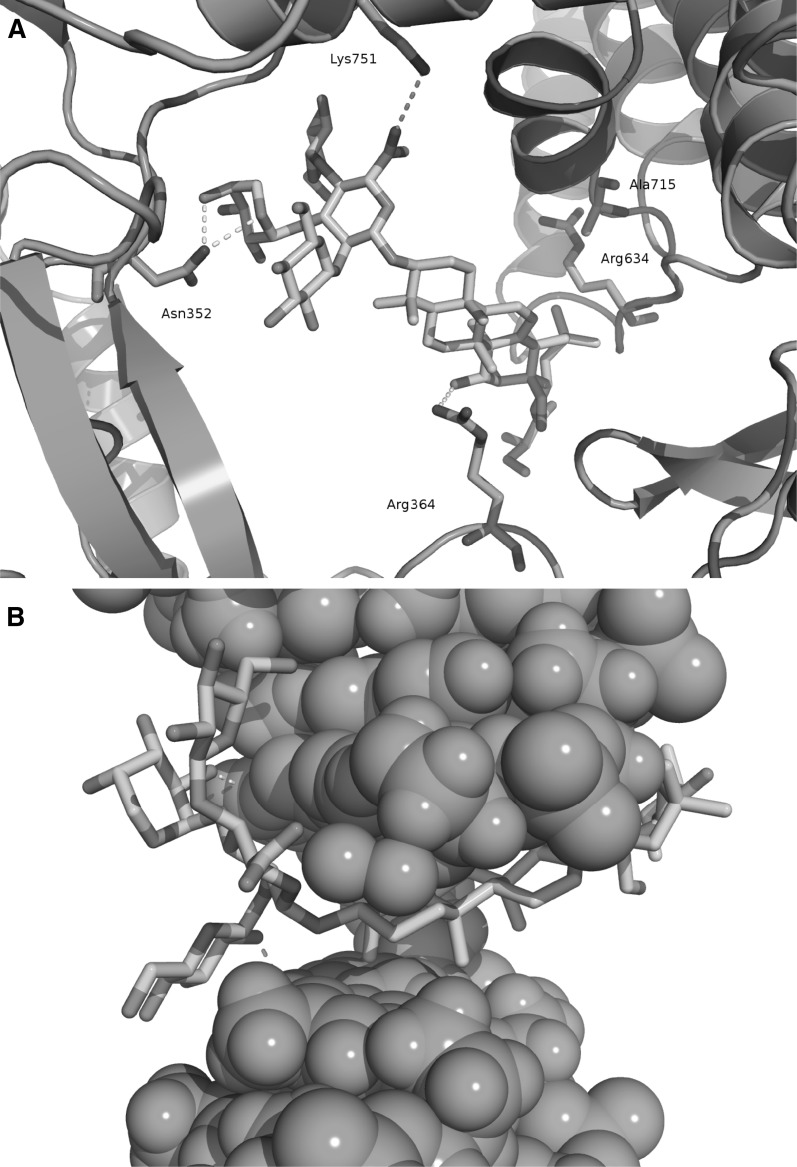
Computational prediction of binding mode of the most active compound (**8**) to the topoisomerase I–DNA complex. **A** Interactions between compound **8**, shown as *sticks*, and topoisomerase I, shown as cartoon in *cyan*. The interacting residues are also shown as *sticks*. Compound **8** forms hydrogen bonds to the residues Asn352, Arg364 and Lys751, as indicated by* yellow dotted lines*. The molecule forms also hydrophobic interactions with the side chains of Arg364, Arg634 and Ala715. **B** Interactions between compound **8** and DNA, shown as *green spheres*. Apart from hydrophobic interactions with the DNA, also two hydrogen bonds to the DNA bases at the cleavage site are formed

Up to date, the binding mode of saponins and structurally similar compounds acting on topoisomerase I has not been determined. To investigate whether triterpene saponins exhibit a similar binding mode as camptothecin, an interfacial inhibitor interacting with the DNA (via stacking) and protein at the DNA cleavage site [23], molecular docking predictions of camptothecin and all investigated saponins were performed. In contrast to camptothecin and other known interfacial topoisomerase inhibitors, triterpene saponins are comparably large molecules with a molecular weight higher than 1 kDa. Nevertheless, a blind docking study investigating possible fits into all cavities in the protein-DNA complex revealed that the saponins are capable of fitting into the same cavity as camptothecin, located at the DNA cleavage site. When analyzing the 100 docking runs for each compound, it was noted that the majority of poses were found in a similar orientation as indicted in Fig. 2 for the most active compound (**8**). Thus, all triterpene saponins were predicted to exhibit a similar binding mode. Although, due to the absence of aromatic rings, saponins, unlike camptothecin, cannot form stacking interactions with the DNA bases, they are capable of crossing the DNA cleavage site and interacting with the DNA via hydrophobic contacts and hydrogen bonds. Furthermore, interactions are also observed between the compounds and the protein residues in the proximity of the cleavage site. In particular, the most active compound (**8**) showed hydrogen bonds to the residues Arg364, Asn352 and Lys751 (Fig. 2), the former two also interacting with other known topoisomerase interfacial inhibitors [23–25]. However, the small differences in topoisomerase I IC_50_ values could not be explained by the presence or absence of specific protein interactions in the docking study. Nevertheless, the investigated compounds showed different hydrogen bond networks with the DNA bases at the cleavage site. Further studies, involving the experimental determination of DNA sequence preferences of the saponin series as well as molecular dynamics simulations, mutagenesis and crystallographic experiments could shed more light on the exact binding mode and the structure–activity-relationship of the described compounds in future.

Finally, it must not necessarily exist a direct connection between these two observed biological activities resp. that the cytotoxic effect was caused (only) by an inhibition of topoisomerase I but it might be an involved mechanism in cytotoxic procedures until further work corroborate or invalidate this point of view.

## Experimental Section

The relaxation assay on human topoisomerase I was carried out by Inspiralis Limited^®^, UK, according to their SOP (standard operating procedure). All compounds were dissolved in DMSO and then diluted to yield concentration ranges from 1 nM to 1 mM. Camptothecin was used as positive control. 1 U (the amount of enzyme required to completely relax the substrate) was incubated with 0.5 µg supercoiled plasmid DNA (pBR322) at 37 °C for 30 min in a 30 µL reaction mixture of 20 mM Tris HCL (pH 7.5), 200 mM NaCl, 0.25 mM EDTA and 5 % glycerol. The reaction was stopped by adding 30 µL chloroform/iso-amyl alcohol (26:1) and 20 µL Stop Dye (40 % sucrose, 100 mM Tris HCl (pH 7.5), 10 mM EDTA, 0.5 μg/mL bromophenol blue) before being loaded on a 0.8 % TAE gel run for 2 h at 80 V. Then bands were visualized by ethidium staining for 10 min and analyzed by documentation equipment (Syngene, Cambridge, UK), gel scanning software (Syngene, GeneTools) and SigmaPlot version 12.3. All assays were carried out in a final DMSO concentration of 10 % and were performed twice.

To predict a possible mode of action, molecular docking studies were performed using GOLD Suite 5.2 (CCDC, UK) with settings optimized in previous studies [24, 25]. Briefly, the crystal structure of the topotecan-topoisomerase I-DNA complex (PDB: 1K4T [26]) was chosen for docking because of the best resolution among the available structures. Rigid docking (100 runs) was performed using the ChemPLP scoring function, a population size of 100 and a maximum of 10^6^ operations. To investigate all possible binding modes, a blind docking was performed, covering all protein cavities. Docking poses were analyzed based on their orientation, the interactions formed with the topoisomerase–DNA complex as well as the docking scores. The root-mean-square deviation between coordinates of all poses of each compound were calculated and used for hierarchical clustering. The largest cluster at a clustering distance of 3 Å was investigated further. With the chosen settings, it was possible to re-dock camptothecin into an orientation similar to the crystal structure pose [23, 24].
